# PROX1 is a predictor of survival for gliomas WHO grade II

**DOI:** 10.1038/bjc.2011.162

**Published:** 2011-05-10

**Authors:** T Elsir, M Qu, S G Berntsson, A Orrego, T Olofsson, M S Lindström, M Nistér, A von Deimling, C Hartmann, D Ribom, A Smits

**Affiliations:** 1 Department of Oncology-Pathology, Karolinska Institutet, CCK R8:05, Karolinska University Hospital, S-17176 Stockholm, Sweden; 2 Department of Neuroscience, Neurology, Uppsala University, S-75185 Uppsala, Sweden; 3 National Board of Forensic Medicine, S-75140 Uppsala, Sweden; 4 Department of Neuropathology, Institute of Pathology, Ruprecht-Karls-University Heidelberg, and Clinical Cooperation Unit Neuropathology, German Cancer Research Center, 69120 Heidelberg, Germany

**Keywords:** low-grade glioma, astrocytoma, oligodendroglioma, PROX1, prognosis, survival

## Abstract

**Background:**

The clinical course of World Health Organisation grade II gliomas remains variable and their time point of transformation into a more malignant phenotype is unpredictable. Identification of biological markers that can predict prognosis in individual patients is of great clinical value. PROX1 is a transcription factor that has a critical role in the development of various organs. PROX1 has been ascribed both oncogenic and tumour suppressive functions in human cancers. We have recently shown that PROX1 may act as a diagnostic marker for high-grade gliomas. The aim of this study was to address the prognostic value of PROX1 in grade II gliomas.

**Methods:**

A total of 116 samples were evaluated for the presence of PROX1 protein. The number of immunopositive cells was used as a variable in survival analysis, together with established prognostic factors for this patient group.

**Results:**

Higher PROX1 protein was associated with poor outcome. In the multivariate analysis, PROX1 was identified as an independent factor for survival (*P*=0.024), together with the presence of mutated isocitrate dehydrogenase 1 R132H protein, and with combined losses of chromosomal arms 1p/19q in oligodendrocytic tumours.

**Conclusion:**

PROX1 is a novel predictor of survival for grade II gliomas.

Adult low-grade gliomas (LGG) are poorly circumscribed diffusely infiltrative brain tumours localised mainly in the cerebral hemispheres. They affect otherwise healthy individuals with an average age of ∼40 years at the time of diagnosis. Low-grade gliomas in adults are classified as grade II gliomas according to the World Health Organisation (WHO) classification of brain tumours and consist mainly of astrocytomas, oligodendrogliomas and oligoastrocytomas (Louis *et al*, 2007). The median survival for LGG is 5–10 years, but clinical outcome varies considerably (Lote *et al*, 1997). For some patients the disease has an indolent course for many years, whereas others experience rapid tumour progression from the time of diagnosis. Treatment is unsatisfactory and there is no cure for LGG (Soffietti *et al*, 2010). There is consensus but no evidence that maximal surgical resection prolongs survival in LGG (Keles *et al*, 2001; Duffau, 2009). A large randomised trial designed to define the optimal timing of radiotherapy showed a prolonged symptom-free survival for patients with LGG receiving adjuvant radiotherapy compared with those irradiated at progression, but no difference in overall survival (Karim *et al*, 2002; van den Bent *et al*, 2005).

For optimal patient management, clinical parameters with an impact on survival have to be taken into account (Pignatti *et al*, 2002; Soffietti *et al*, 2010). Negative prognostic factors for survival are old age, astrocytoma histology, neurological deficit at presentation, large tumour diameter, contrast enhancement and tumours crossing midline (Pignatti *et al*, 2002). The presence of two or less unfavourable factors identifies low-risk groups, and for these low-risk patients it is probably a good strategy to defer radiotherapy until tumour progression (Pignatti *et al*, 2002). Many treatment decisions, however, remain unsolved. The clinical management of patients with LGG will benefit from prognostic and predictive biomarkers that can guide therapeutic decisions. One such example is the combined loss of chromosomal arms 1p/19q, the molecular hallmark of oligodendrogliomas and a strong prognostic and predictive marker in these tumours (Ohgaki and Kleihues, 2005).

PROX1 is a transcription factor expressed in the heart, liver, lens, skeletal muscles, pancreas, kidney and in the CNS (Zinovieva *et al*, 1996). The Drosophila homologue of PROX1, prospero, resembles a tumour suppressor gene by preventing neuroblast self-renewal. In the absence of prospero, cells accumulate forming brain tumours (Betschinger *et al*, 2006). It has been suggested that PROX1 has a similar role in human malignancies. Decreased expression of PROX1 was found in hepatocellular carcinomas, sporadic breast cancer and carcinomas of the biliary system (Shimoda *et al*, 2006; Laerm *et al*, 2007; Versmold *et al*, 2007). In contrast, PROX1 was overexpressed in colorectal cancer and found to have a role in promoting cancer progression (Petrova *et al*, 2008). We have recently shown that PROX1 protein is overexpressed in human astrocytic tumours, with highest levels in grade III and IV astrocytomas (Elsir *et al*, 2010). These findings suggest that PROX1 may act as a prognostic factor in human gliomas. In the present study, we have studied the presence of PROX1 protein in correlation with patient survival in a cohort of patients with LGG.

## Materials and methods

### Tumour samples

Tumour samples with primary histopathological diagnosis of supratentorial WHO grade II glioma were collected from patients aged ⩾16 years operated between January 1982 and December 1999 at neurosurgery, Uppsala University Hospital. The ethics committee approved the study protocol and recruitment of patients was based on informed consent. A total number of 152 paraffin-embedded tumour blocks were identified and pre-examined by a neuropathologist (TO) to verify representative tumour material. Samples were then sectioned and evaluated by the review neuropathologist (AO), who had not been involved in the primary diagnosis. Tumours were classified as astrocytomas (including gemistocytic astrocytomas), oligodendrogliomas and oligoastrocytomas grade II according to the WHO classification of brain tumours (Louis *et al*, 2007). Representative areas in the tumour bulk, consisting of predominantly tumour cells (>80%) compared with normal cells, were marked on each slide. Pilocytic astrocytomas, gangliogliomas, pleomorphic xanthoastrocytomas and tumours with signs of anaplasia were excluded (*n*=23). Nine samples were of poor technical quality, leaving 120 tumours.

### Collection of clinical data

A retrospective chart review was performed for all 120 cases. In four cases, medical files were incomplete and clinical data could not be evaluated, and a total of 116 cases were included in the present study. The following data were collected from the patient records and from CT or MRI scans of the brain: time point of first symptoms, patient age at disease onset, date of operation, date of death, tumour size, tumour location (specific lobe and (sub)cortical or central location), the presence of contrast enhancement, preoperative performance status according to Karnofsky Performance Status, date and extent of diagnostic surgery (biopsy, subtotal or gross total resection). The extent of tumour resection was based on postoperative CT scans or on the surgeon's operative notes.

Survival was defined as the time point between first symptoms and date of death or end of the study (20 September 2009). Data concerning time of death and the cause of death were collected from central health authorities (the National Cause of Death Register Data).

### Validation of PROX1 antibodies

For expression analysis, we evaluated two commercially available anti-PROX1 antibodies, one goat (R&D Systems, Minneapolis, MN, USA; diluted 1 : 100) and one rabbit polyclonal (ab11941; Abcam, Cambridge, UK; diluted 1 : 100), raised against the N-terminus and C-terminus of PROX1, respectively. Validation of the sensitivity and specificity of the antibodies was tested previously (Elsir *et al*, 2010). In summary, we tested the antibodies by using SW480 colon carcinoma cells, known to express PROX1, and U2OS osteosarcoma cells, used as a negative control (Petrova *et al*, 2008). Immunostaining by the immunofluorescence technique showed negative staining for the U2OS cells and an identically strong nuclear PROX1 staining in SW480 cells for both antibodies. We then used PROX1 siRNA to knock out gene expression from the SW480 cells and overexpressed PROX1 cDNA in the negative cell line U2OS. Immunostaining subsequent to these techniques further confirmed the specificity of the antibodies, in agreement with published findings on PROX1 protein (Petrova *et al*, 2008).

### PROX1 immunostaining

Immunostaining with anti-PROX1 antibodies was performed on formalin-fixed and paraffin-embedded sections using the Ventana Discovery Automated Stainer (Ventana Medical Systems, Tucson, AZ, USA), following Vendor's instructions. Deparaffinisation took place in the Ventana machine and the heat-induced epitope retrieval incubation was performed in Tris-Borate EDTA buffer pH 8.0. Primary antibody was a goat anti-PROX1 antibody (R&D System), purchased commercially and diluted 1 : 100 in 1% BSA, 0.1% Tween-20 in PBS. A streptavidin-biotin horseradish peroxidase based DAB kit provided by Ventana was used for detection of immunoreactivity. Secondary antibody was a polyclonal biotinylated rabbit anti-goat (Dako E0466, Dako Sweden AB, Stockholm, Sweden) diluted at 1 : 500 in Ab-diluent (Ventana Medical Systems). Sections were counterstained with haematoxylin. Finally, slides were subjected to graded ethanol rinses, cleared in Xylene and mounted in Pertex. Sections from a glioblastoma case, known to express high levels of PROX1, were added as a positive control in each Ventana run.

### Detection of mutated IDH1 R132H protein

Mutated isocitrate dehydrogenase 1 (IDH1) R132H protein was detected using a monoclonal mouse antibody targeting the mutated IDH1 R132H protein, known as mIDH1R132, as described previously (Capper *et al*, 2009).

### Evaluation of immunostaining

Each section stained by anti-PROX1 antibodies was examined under × 100–400 magnifications using an Olympus light microscope (UPMTVC, Olympus, Tokyo, Japan) and a coupled Leica camera (DFC320, Leica Microsystems GmbH, Wetzlar, Germany). Representative tumour areas, marked on each section by the neuropathologist, were used for counting PROX1 immunopositive cells. Areas with the highest density of immunopositive cells were chosen. At least 200 cells were counted for each section and the proportion of PROX1 immunopositive tumour cells was calculated. The counts were performed independently by two of the authors (TE and AS). All cases were anonymised before scoring and the authors had no access to the clinical data before counting.

Both authors (TE and AS) also evaluated immunostainings by the MIB-1 antibody, which was performed as part of the diagnostic review. The MIB-1 antibody recognises the Ki-67 protein, which is strongly associated with cell proliferation (Johannessen and Torp, 2006). Samples with the highest amount of Ki-67 immunopositive tumour cells were identified and separated from samples with lower amount of positive cells.

Evaluation of immunostaining with mIDH1R132 was performed by one of the neuropathologists (AvD) as previously reported, and samples were classified as either positive or negative for mutated IDH1 protein (Capper *et al*, 2009).

### Fluorescent *in situ* hybridisation

Fluorescent *in situ* hybridisation analysis (FISH) on paraffin sections to study losses of the chromosomal arms 1p and 19q was performed as described previously (Broholm *et al*, 2008). The commercially purchased probes used for hybridisation were Zytolight SPEC 1p36/1q25 and 19q13/19p13 dual colour probes (Nordic BioSite, Täby, Sweden). Slides were assessed under a fluorescence microscope (Olympus BX50, Olympus Deutschland GmbH, Hamburg, Germany), and a minimum of 100 non-overlapping nuclei of cells located in the tumour bulk were evaluated for numbers of green (reference probe) and red signals (target probe) in each hybridisation. A tumour was considered deleted when >50% of the nuclei harboured only one red signal of the target probe and the two green signals of the reference probe.

### Statistical analysis

The statistical calculations were performed in JMP, version 5.0.1a (SAS Institute Inc., Cary, NC, USA). Survival curves were plotted according to the Kaplan–Meier method (product-limit method) and the log-rank probability test (Mantel–Cox) estimated the prognostic value of the PROX1 expression in the univariate analysis. The Cox proportional hazard model was used to calculate the impact of PROX1 expression in the multivariate analysis together with established prognostic factors. Established clinical prognostic factors for LGG were used in the model (Pignatti *et al*, 2002), as well as contrast enhancement, extent of resection, radiotherapy and the molecular markers Ki-67, mutated IDH1 R132H protein and combined loss of 1p/19q. The level for confounders to be removed from the model when adjusted for variables already in the model (p-to-remove) was set to >0.1. The natural logarithm (ln) cumulative hazard plots were made to confirm the assumption of the proportional hazard functions. The significant prognostic factors in the stepwise model were also analysed as products to minimise the possibility of interaction.

## Results

### Patient characteristics

Clinical characteristics of the 116 patients are presented in Table 1. The mean age at the time of disease onset was 40 years. Patients mostly presented with seizures, others with headaches and cognitive disturbances. The majority of patients had duration of symptoms <6 months and a good preoperative clinical status at the time of surgery. Most common type of surgery was subtotal tumour resection. Almost all patients had radiotherapy. Chemotherapy was given in 22 cases and ruled out in 7 cases, but the exact number of patients receiving chemotherapy could not be evaluated due to insufficient follow-up data at local hospitals (not shown).

### Tumour characteristics

The characteristics of the tumours are presented in Table 2. Histopathological diagnoses comprised astrocytomas (including gemistocytic), oligoastrocytomas and oligodendrogliomas WHO grade II. Most tumours were located in the frontal lobe. Others had temporal, parietal, occipital or central location, or involved more than one lobe. The majority of tumours was <6 cm in diameter, showed no contrast enhancement and did not cross midline structures.

### Molecular characteristics

Table 3 shows the molecular tumour profiles, summarising the results of PROX1 protein expression, mutated IHD1 R132 protein, combined losses of chromosomal arms 1p and 19q, that is, loss of heterozygosity (LOH) 1p/19q, and Ki-67.

#### Detection of PROX1 protein

**Figure 1:**
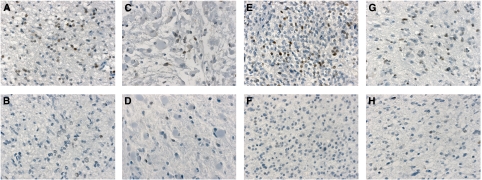
PROX1 protein expression in WHO grade II gliomas. Immunohistochemical staining of PROX1 (brown colour) demonstrating the variability in the amount of PROX1 immunopositive cells between the different tumours samples. (**A** and **B**) Fibrillary astrocytoma with a relatively high respectively low number of PROX1 expressing tumour cells. (**C** and **D**) Gemistocytic astrocytoma with a relatively high respectively low number of PROX1 expressing tumour cells. (**E** and **F**) Oligodendroglioma with a relatively high respectively low number of PROX1 expressing tumour cells. (**G** and **H**) Oligoastrocytoma with a relatively high respectively low number of PROX1 expressing tumour cells.

PROX1 was detected in all 116 cases and manifested as a well-defined nuclear staining. There was an evident variability between the numbers of PROX1 immunopositive tumour cells in the different samples (Figure 1). We also found some intra-tumoral variability in distribution of PROX1-positive cells, as well as a minor variation in staining intensity between different tumour cells within the same sample. We counted only clearly positive nuclear stained cells and consequently counted cells in tumour areas with highest number of immunopositive cells. Results of the two independently performed counts showed agreement (i.e., ⩽10% difference in the proportion of positive tumour cells assessed by the two observers) for 89 samples. For 27 cases, the inter-observer variability was >10%. We recounted these samples and grouped them into three categories (<10% positive cells, 10–30% positive cells and >30% positive cells). This way we came to a consensus for all samples.

The mean percentage of positive cells for all 116 samples was 15%. Oligoastrocytomas showed a mean of 17.7%, astrocytomas of 16.5%, gemistocytic astrocytomas of 10.6% and oligodendrogliomas of 12.5% PROX1 immunopositive tumour cells. The proportional distribution of PROX1 immunopositive tumour cells was found similar for all three histological groups. Most samples showed PROX1 protein in <10% of the tumour cells, the remaining had between 10 and 30% or >30% immunopositive tumour cells (Table 2).

#### Detection of mutated IDH1 R132 protein

Ninety tumour samples were found to stain for the mutated IDH1 R132H protein (Table 3). In general, most cells stained positively for mIDH1R132 in tumours that harboured the mutated IDH1 R132H protein. It has been reported that this antibody has a high specificity to tumour cells (Capper *et al*, 2009). Therefore, cells within IDH1-positive tumours that are not stained by mIDH1R132 are considered to be non-tumour cells.

#### Analysis of LOH 1p/19q by FISH

Twenty-five oligodendrogliomas showed combined losses of chromosomal arms 1p/19q. One oligodendroglioma had 1p deletion only without loss of 19q, and was considered as LOH 1p/19q negative. Eight oligoastrocytomas showed combined 1p/19q loss. None of the astrocytomas in our series was found with this chromosomal abnormality (Table 3).

### Univariate survival analysis

In all, 102 of the 116 identified patients (87%) died at the end of the study. The median follow-up time for the 14 censored patients was 14.5 years (range 10.0–22.5 years). The 5-year survival for the whole patient sample was 58% and the 10-year survival was 32%. Median survival was 6.2 years.

**Figure 2:**
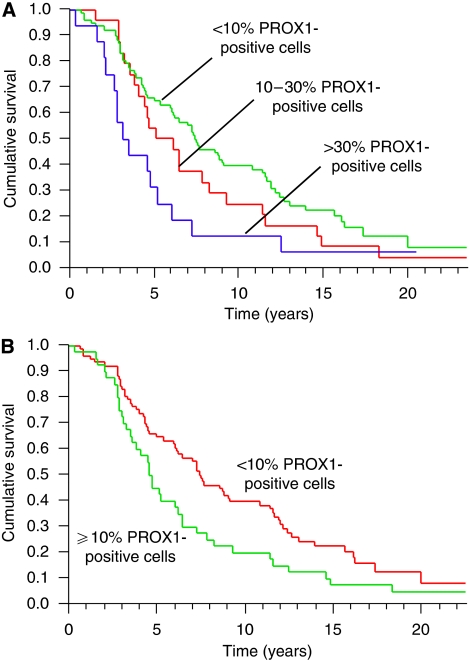
(**A**) Kaplan–Meier estimates of postoperative survival by the variable PROX1 protein expression for all samples with <10%, 10–30% and >30% immunopositive tumour cells. (**B**) Kaplan–Meier estimates of survival by the variable PROX1 protein expression after dichotomisation of PROX1 into <10% and ⩾10% immunopositive tumour cells (*P*=0.0183, log-rank test).

Analysis of the prognostic significance of PROX1 using the Kaplan–Meier model showed that high PROX1 expression (>30% of the tumour cells) correlated with shorter overall survival, compared with lower PROX1 expression (10–30%, respectively, <10% of the tumour cells) (Figure 2A). Dichotomisation of the variable PROX1 expression into ⩽10% and >10% positive cells showed that PROX1 expression >10% was associated with statistically significant shorter overall survival (*P*=0.0183, log-rank test) (Figure 2B).

### Multivariate survival analysis (*n*=116)

Due to limited sample size in relation to the large number of established prognostic markers, the stepwise exclusion of variables was used to achieve a model with as few variables as possible in the multivariate analyses. Both models (the ‘full model’ and the ‘stepwise model’) are presented in Table 4.

#### Clinical variables and survival

Radiotherapy as a variable had an inverse relation to survival that was considered to be a selection bias, and was therefore removed from the Cox model. The variables tumour crossing midline, contrast enhancement and astrocytoma histology were all significant factors for poor overall survival in the multivariate analysis. The variables higher age at disease onset and large tumour size were not associated with shorter overall survival.

#### Molecular variables and survival

Multivariate analysis using the Cox's proportional hazard model identified the variable PROX1 expression as an independent predictor for poor overall survival, in the full model (*P*=0.0310) as well as in the stepwise model (*P*=0.0237) (Table 4). The variable IDH1 mutation was identified as an independent predictor for longer overall survival in the full model. Statistical analysis of Ki-67 expression (≅4% *vs* <4%) did not reveal any significant difference in total survival between the two groups. Since all astrocytomas had wild-type 1p/19q status, the impact of LOH 1p/19q as a prognostic factor was not analysed in the whole sample, but used in a separate Cox model including tumours with oligodendrocytic histology only (Table 5).

#### Survival analysis in oligodendrocytic tumours (*n*=73)

Multivariate analysis using the Cox's proportional hazard model identified the variable PROX1 expression as an independent predictor for poor survival in the full model (*P*=0.0275), as well as in the stepwise model (*P*=0.0269) (Table 5). As shown, the clinical variables of younger age at disease onset together with the absence of contrast enhancement had significantly favourable impact on survival in this group. The variable LOH 1p/19q was identified as a strong independent predictor for longer survival in the full model, as well as in the stepwise model. The variable IDH1 mutation was not identified as an independent predictor for longer survival in the oligodendrocytic tumour group.

## Discussion

In the present study, we investigated the presence of PROX1 protein and its association with survival in a series of gliomas WHO grade II. We found a correlation between high number of PROX1 immunopositive tumour cells and poor outcome of patients. PROX1 emerged as an independent marker for survival in the multivariate analysis, together with established molecular prognostic factors such as LOH 1p/19q and mutated IDH1 R132H protein. Our results suggest that PROX1 protein expression is a new molecular marker for survival in patients with grade II gliomas.

Low-grade gliomas in adults have a highly variable course of disease. The most common presenting symptoms are epileptic seizures. Epilepsy is often the only symptom during early disease (Smits and Duffau, 2011). It is well known though that LGG grow continuously and lead to neurological disability and ultimately to death (Mandonnet *et al*, 2003; Schiff *et al*, 2007; Rees *et al*, 2009). Clinical parameters correlating with prognosis such as tumour histology, patient age, performance status, tumour size and the presence of contrast enhancement have been used to guide treatment decisions (Pignatti *et al*, 2002; Soffietti *et al*, 2010). In essence, we confirmed the prognostic impact of these clinical parameters in the present study, although evaluation by retrospective review of medical files is not optimal. For example, chemotherapy (mostly used as second-line treatment in patients with oligodendrogliomas) could not be included as a variable in the survival analysis due to missing data. Also, contrast enhancement was found more often than expected but we could not discriminate nodular from non-nodular type of contrast enhancement. Only the nodular type of contrast leaking into the tumour has been associated with poor outcome (Pallud *et al*, 2009).

We found a strong correlation between favourable outcome and LOH 1p/19q in oligodendrocytic tumours, confirming the importance of this biomarker for survival. All astrocytomas in our sample were found to be wild type for 1p and 19q, which is consistent with previous studies on LGG (Pouratian and Schiff, 2010). In addition, the presence of mutated IDH R132H protein was identified as an independent marker for longer survival in our study sample (Yan *et al*, 2009). Mutations in *IDH1* and the clinical implications of this biomarker for gliomas have received considerable attention lately (Von Deimling *et al*, 2011). Heterozygous point mutations in codon 132 are frequently present in LGG, anaplastic gliomas and secondary glioblastomas, that is, highly malignant tumours that evolve from previously confirmed LGG. In the vast majority of cases, *IDH1* mutations affect codon 132 and in 93% of all cases they are of the R132H type (Von Deimling *et al*, 2011). The development of an IDH1 R132H mutation-specific antibody suitable for immunohistochemistry has largely facilitated detection of mutated IDH1 protein in clinical practice (Capper *et al*, 2009). As both PROX1 protein expression and mutated IDH1 R132 protein were identified as prognostic factors in our study, we searched for a possible correlation between the two biomarkers but did not find any evidence for this (data not shown).

Statistical analysis of Ki-67 expression, with a cutoff of 4%, was not identified as a prognostic marker in our study. This finding is also consistent with previous reports. MIB-1 labelling has been shown particularly useful for differentiating between diffuse and anaplastic astrocytomas, but there is considerable overlap between the labelling index in these different tumours and diverging cutoff values have been proposed (Johannessen and Torp, 2006).

PROX1 is a transcription factor and a key player in the development of the lymphatic system (Wigle and Oliver, 1999). In the mammalian CNS, PROX1 regulates gene expression and is involved in neurogenesis (Wigle *et al*, 1999; Misra *et al*, 2008). Inactivation of PROX1 in the developing eye lens leads to the downregulation of the cell cycle inhibitors p27 and p57 and deregulation of E-cadherin (Wigle *et al*, 1999). Recently, we reported on the expression patterns of PROX1 in astrocytic gliomas. We found overexpression of PROX1 protein in high-grade compared with low-grade gliomas and demonstrated that the percentage of tumour cells expressing PROX1 correlated with the malignancy grade of the tumour, which prompted further studies with focus on the expression of PROX1 in relation to clinical parameters and patient survival (Elsir *et al*, 2010).

We chose to evaluate PROX1 protein levels by scoring cells as either positive or negative, based on our findings of relatively little variation in staining intensity. In our previous report, we graded immunostaining as strong positive, weak positive or negative. In that study, samples from high-grade gliomas showed a larger variation in staining intensity. The majority of the LGG in the present study contained relatively few PROX1 expressing cells, consistent with our previous findings that PROX1 protein expression was generally higher in high-grade than in low-grade gliomas. One could hypothesise that those tumours in the present study with a higher proportion of immunopositive cells represent a more advanced, less differentiated phenotype than their counterparts with relatively low PROX1 expression. Although still consistent with the histopathological diagnosis of WHO grade II glioma, such tumours displaying a high proportion of PROX1-positive cells may be further advanced on the evolution to anaplastic gliomas compared with tumours with few PROX1 expressing tumour cells. Although the histology of LGG is far less heterogeneous than in high-grade gliomas, LGG are likely to represent clinically different stages of evolution ranging from indolent to more aggressive variants. To be able to foresee early progression of these tumours would help extensively in clinical practice, where more aggressive treatment should be postponed for patients with low risk for tumour progression (Piepmeier, 2009; Soffietti *et al*, 2010)

Within the limitations of the study design, we made an effort to provide reproducible results and to avoid flaws that commonly occur in retrospective data collection. Samples were identified by searching clinical records of patients operated between 1982 and 1999, and were selected on the basis of the availability of good quality paraffin-embedded tumour blocks only. Samples were reviewed by independent neuropathologists who marked representative tumour areas on the back of the slides to be used for evaluation of immunostaining. Follow-up time of the cohort was long, with a minimum of 10 and up to 25 years.

In conclusion, the present findings support our previous work showing that higher PROX1 protein expression in gliomas reflects a more malignant phenotype. Our study also promotes PROX1 as a prognostic marker for WHO grade II gliomas. Further studies on the functional role of PROX1 as a biomarker in gliomas are warranted.

## Figures and Tables

**Table 1:** Clinical characteristics of the patients with WHO grade II gliomas included in the study (*n*=116)

**Parameter**	**Number of patients (%)**
*Gender*	
Male	76 (65.5)
Female	40 (34.5)
	
*Age*	
Mean (years)	40.0 years
<20	3 (2.6)
20–29	22 (19.0)
30–39	39 (33.6)
40–49	23 (19.8)
50–59	17 (14.7)
⩾60	12 (10.3)
	
*Presenting symptoms*	
Seizures	95 (81.9)
Headache	8 (6.9)
Cognitive dysfunction	6 (5.2)
Others	7 (6.0)
	
*Preoperative KPS*	
>80	78 (67.3)
⩽80	38 (32.7)
	
*Duration of symptoms before operation (months)*	
⩽6	72 (62.1)
7–12	12 (10.3)
13–24	12 (10.3)
>24	20 (17.3)
	
*Surgery*	
Biopsy only	37 (31.9)
Subtotal resection	54 (46.5)
Gross total resection	25 (21.6)
	
*Radiotherapy*	
Yes	104 (89.7)
No	11 (9.5)
Uncertain	1 (0.8)

Abbreviations: WHO=World Health Organisation; KPS=Karnofsky Performance Status.

**Table 2:** Tumour characteristics of the WHO grade II gliomas included in the study (*n*=116)

**Parameter**	**Number of patients (%)**
*Location*	
Frontal	56 (48.3)
Temporal	17 (14.7)
Parietal	4 (3.4)
Occipital	1 (0.9)
Central	2 (1.7)
Corpus callosum	1 (0.9)
Multi-lobular	35 (30.1)
	
*Histology*	
Astrocytoma	32 (27.6)
Gemistocytic astrocytoma	11 (9.5)
Oligoastrocytoma	34 (29.3)
Oligodendroglioma	39 (33.6)
	
*Largest diameter (cm)*	
<6	84 (72.4)
⩾6	32 (27.6)
	
*Contrast enhancement*	
Yes	52 (44.8)
No	64 (55.2)
	
*Crossing midline*	
Yes	34 (29.3)
No	82 (70.7)
	
*PROX1 expression* (% *positive cells)*	
<10%	76 (65.5)
10–30	24 (20.7)
>30	16 (13.8)
	
*Mutated IDH1 protein*	
Yes	90 (77.6)
No	26 (22.4)
	
*LOH 1p/19q*	
Yes	33 (28.4)
No	83 (71.6)

Abbreviations: IDH1=isocitrate dehydrogenase 1; LOH=loss of heterozygosity.

**Table 3:** Molecular profiles of the tumour samples (*n*=116)

	**Astro (*n*=32)**	**Gemistocytic astro (*n*=11)**	**Oligoastro (*n*=34)**	**Oligo (*n*=39)**	**Total (*n*=116)**
⩾10% PROX1-positive cells	11	3	15	11	40
Mutated IDH1 R132 protein	22	9	28	31	90
LOH 1p/19q	0	0	8	25	33
Ki-67≅4%	5	1	5	12	23

Abbreviations: IDH1=isocitrate dehydrogenase 1; LOH=loss of heterozygosity.

**Table 4:** Cox's proportional hazard model estimating the prognostic impact of PROX1 expression and of established prognostic factors for LGG on survival (*n*=116)

		**Full model**			**Stepwise model**		
**Prognostic factor**		**Risk ratio**	**Confidence limits**	***P*-value**	**Risk ratio**	**Confidence limits**	***P*-value**
*Clinical variables*							
Age at onset	<40 *vs* ⩾40 years	1.24	0.87–187	0.3167	—	—	—
Performance status	KPS >80 *vs* ⩽80	0.95	0.58–1.60	0.8380	—	—	—
Tumour size	⩾6 *vs* <6 cm	1.37	0.83–2.21	0.2184	—	—	—
Tumour crossing midline	Yes *vs* no	1.71	1.09–2.67	0.0211	1.71	1.11–2.61	0.0161
Contrast enhancement	Yes *vs* no	1.62	1.06–2.47	0.0248	1.52	1.01–2.28	0.0446
Histology	A *vs* OA/O	1.22	0.99–1.51	0.0624	1.22	1.00–1.50	0.0557
Extent of resection	GTR *vs* not	0.75	0.43–1.27	0.2951	—	—	—
							
*Molecular variables*							
Ki-67 expression	<4% *vs* ≅4%	0.93	0.51–1.65	0.8143	—	—	—
IDH1 mutation	Yes *vs* no	0.57	0.35–0.95	0.0313	0.61	0.39–1.02	0.0575
PROX1-positive cells	⩾10% *vs* <10%	1.61	1.04–2.47	0.0310	1.63	1.07–2.45	0.0237

Abbreviations: KPS=Karnofsky performance status; A=astrocytoma; OA=oligoastrocytoma; O=oligodendroglioma; GTR=gross total resection; IDH=isocitrate dehydrogenase; LGG=low-grade gliomas.

Factors removed from the model using the backwards exclusion method (p-to-remove >0.10): Performance status (at step 1, *P*=0.8380), Ki-67 expression (at step 2, *P*=0.8627), age at onset (at step 3, *P*=0.3101), extent of resection (at step 4, *P*=0.2742), tumour size (at step 5, *P*=0.1057).

**Table 5:** Cox's proportional hazard model estimating the prognostic impact of PROX1 expression and of established prognostic factors on survival in patients with oligodendrogliomas and oligoastrocytomas WHO grade II (*n*=73)

		**Full model**			**Stepwise model**		
**Prognostic factor**		**Risk ratio**	**Confidence limits**	***P*-value**	**Risk ratio**	**Confidence limits**	***P*-value**
*Clinical variables*							
Age at onset	<40 *vs* ⩾40 years	2.09	1.14–3.83	0.0177	2.11	1.13–3.85	0.0144
Performance status	KPS >80 *vs* ⩽80	0.76	0.38–1.56	0.4474	—	—	—
Tumour size	⩾6 *vs* <6 cm	1.79	0.91–3.41	0.0892	1.81	0.95–3.37	0.0712
Tumour crossing midline	Yes *vs* no	1.78	0.95–3.27	0.0720	1.76	0.95–3.21	0.0722
Contrast enhancement	Yes *vs* no	2.37	1.32–4.31	0.0040	2.28	1.28–4.09	0.0052
Histology	OA *vs* O	1.39	1.02–1.92	0.0395	1.32	0.98–1.79	0.0683
Extent of resection	GTR *vs* not	0.86	0.40–1.71	0.6689	—	—	—
							
*Molecular variables*							
Ki-67	<4% *vs* ≅4%	1.97	0.90–4.11	0.0883	2.02	0.95–4.07	0.0662
IDH1 mutation	Yes *vs* no	0.72	0.36–1.53	0.3786	—	—	—
LOH 1p/19q	Yes *vs* no	1.62	1.15–2.31	0.0052	1.77	1.31–2.44	0.0002
PROX1-positive cells	⩾10% *vs* <10%	2.00	1.08–3.70	0.0275	1.98	1.08–3.63	0.0269

Abbreviations: KPS=Karnofsky performance status; OA=oligoastrocytoma; O=oligodendroglioma; GTR=gross total resection; IDH=isocitrate dehydrogenase; LOH=loss of heterozygosity; WHO=World Health Organisation.

Factors removed from the model using the backwards exclusion method (p-to-remove >0.10): Extent of resection (at step 1, *P*=0.6689), performance status (at step 2, *P*=0.3997), IDH1 mutation (at step 3, *P*=0.5105).
